# Supplementary figures and images for: Osteopontin on the Dental Implant Surface Promotes Direct Osteogenesis in Osseointegration
Source: Int J Mol Sci. 2022 Jan 18;23(3):1039. doi: 10.3390/ijms23031039 (PMC8835189; doi:10.3390/ijms23031039)

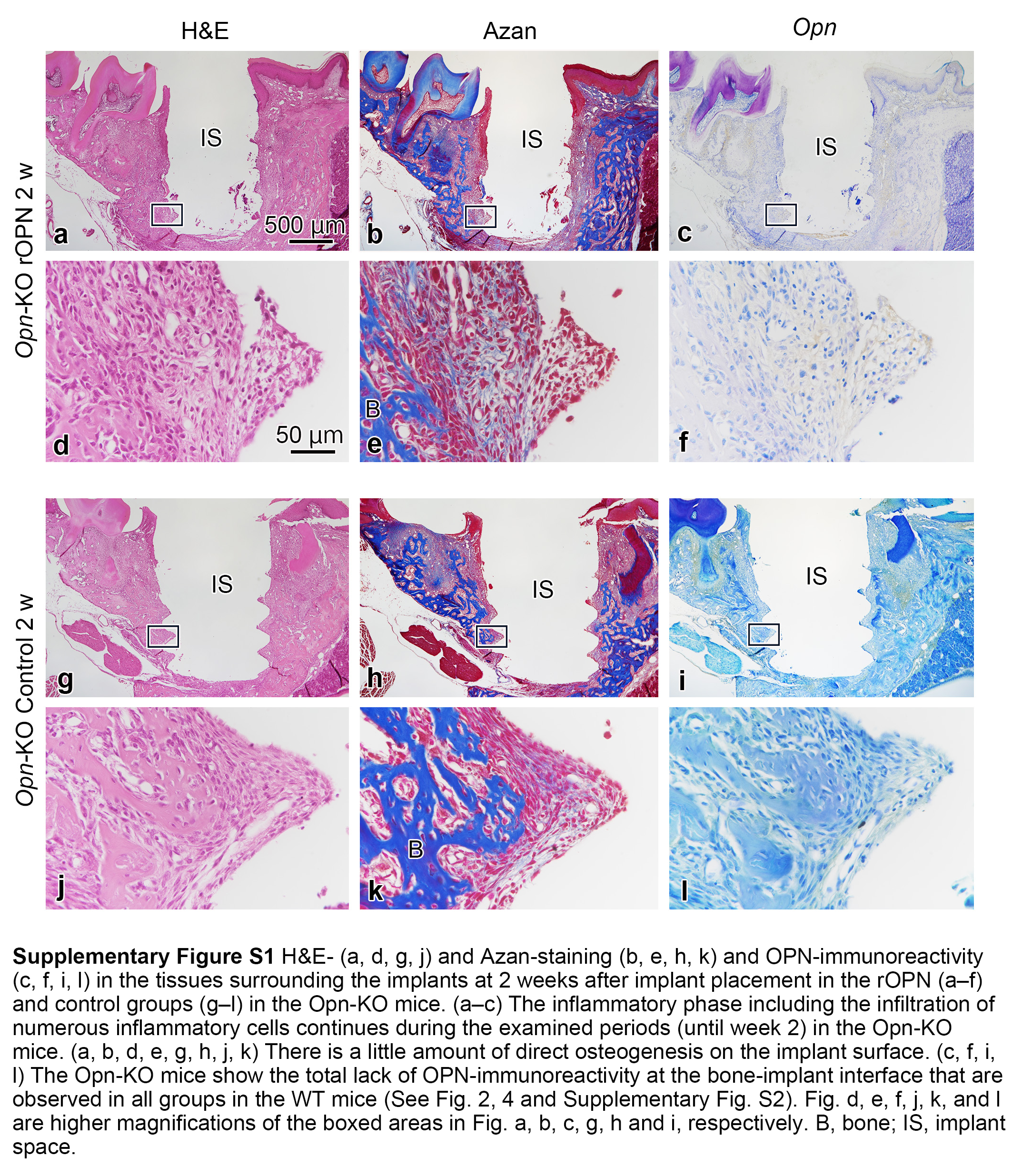

Supplement: Supplementary file 1 [file ijms-23-01039-s001.zip › Figure S1.tif]

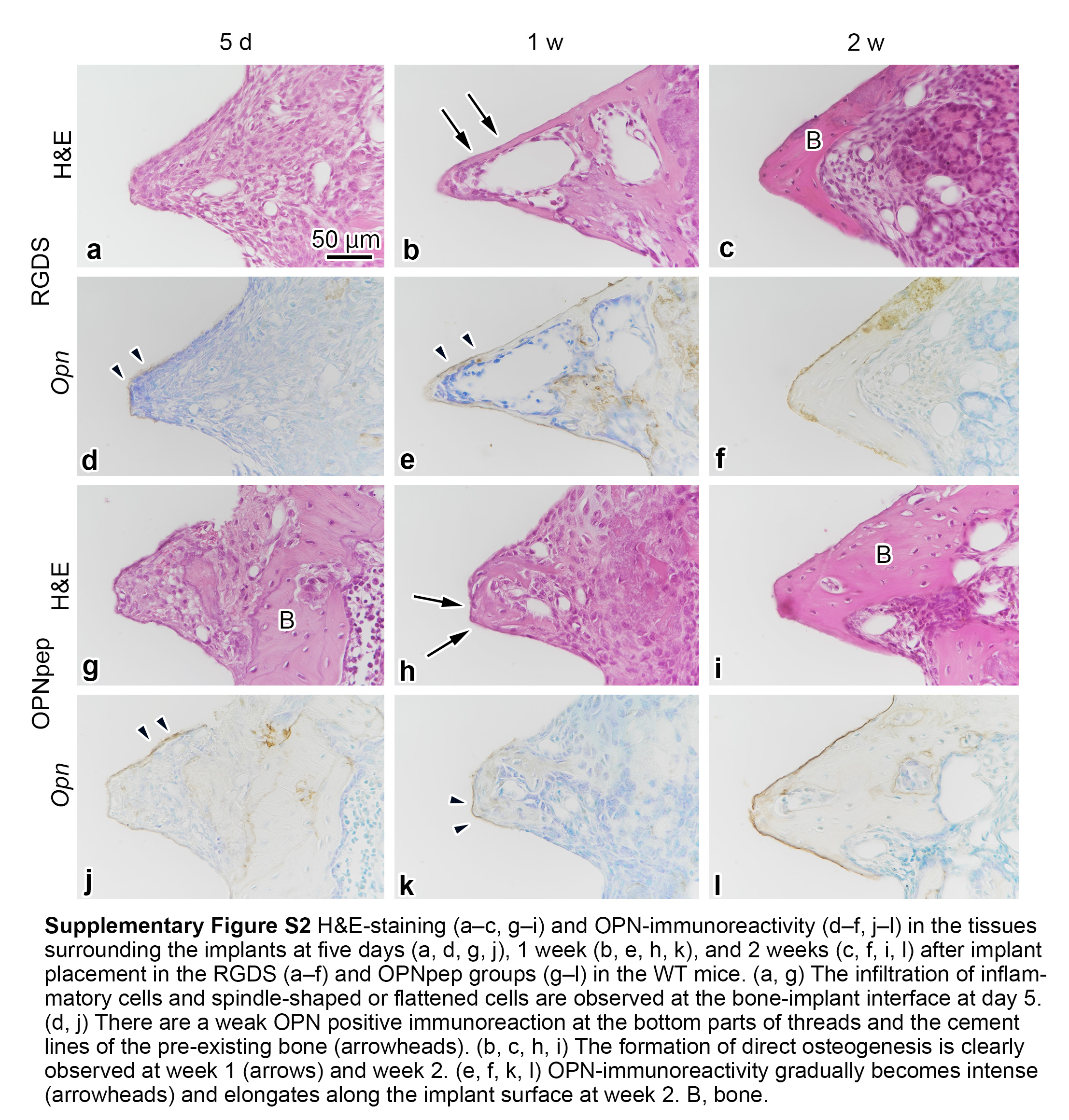

Supplement: Supplementary file 1 [file ijms-23-01039-s001.zip › Figure S2.tif]

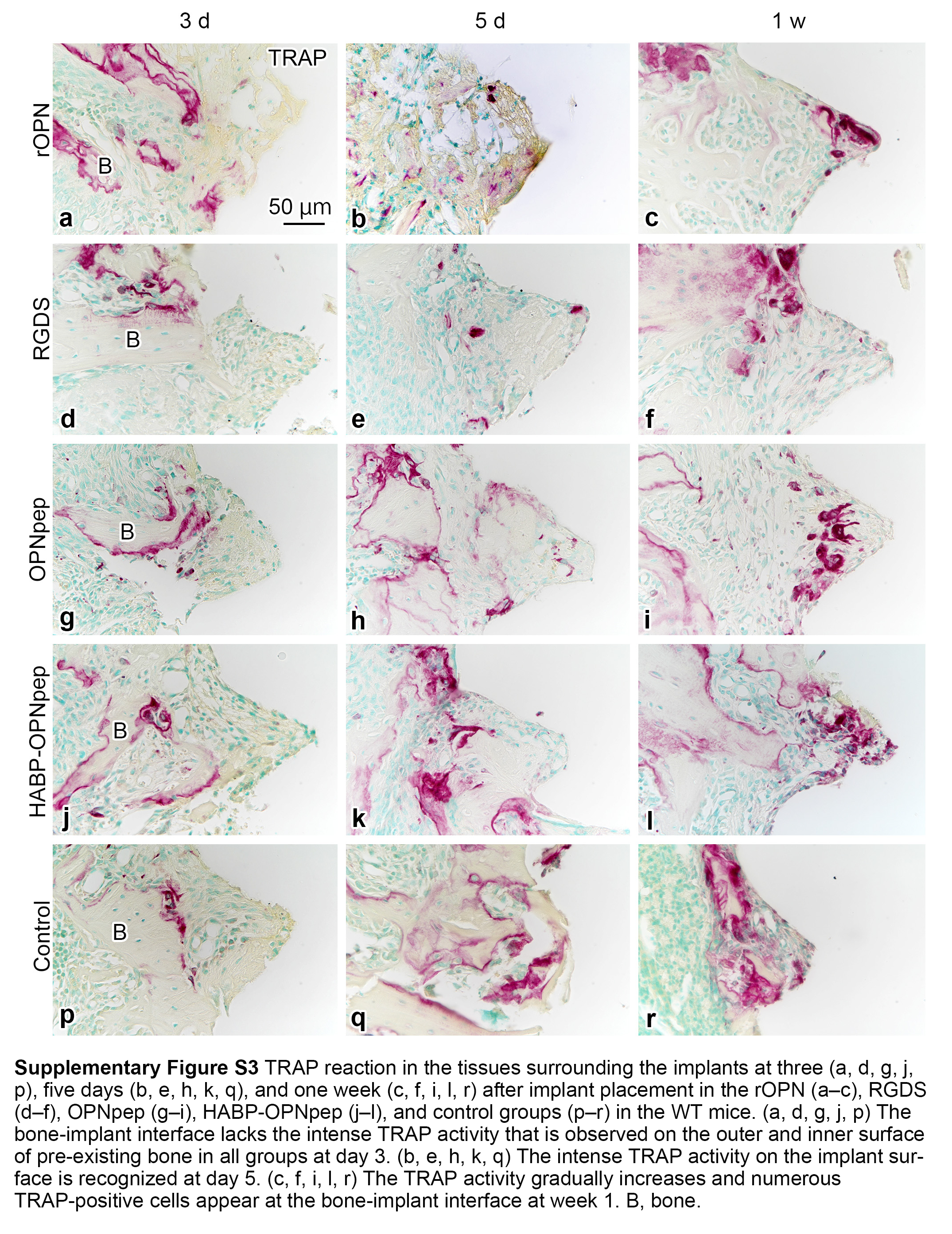

Supplement: Supplementary file 1 [file ijms-23-01039-s001.zip › Figure S3.tif]
